# George Winthrop Sherouse, Ph.D., DABR, FAAPM

**DOI:** 10.1002/acm2.13553

**Published:** 2022-02-08

**Authors:** Jessica B. Clements


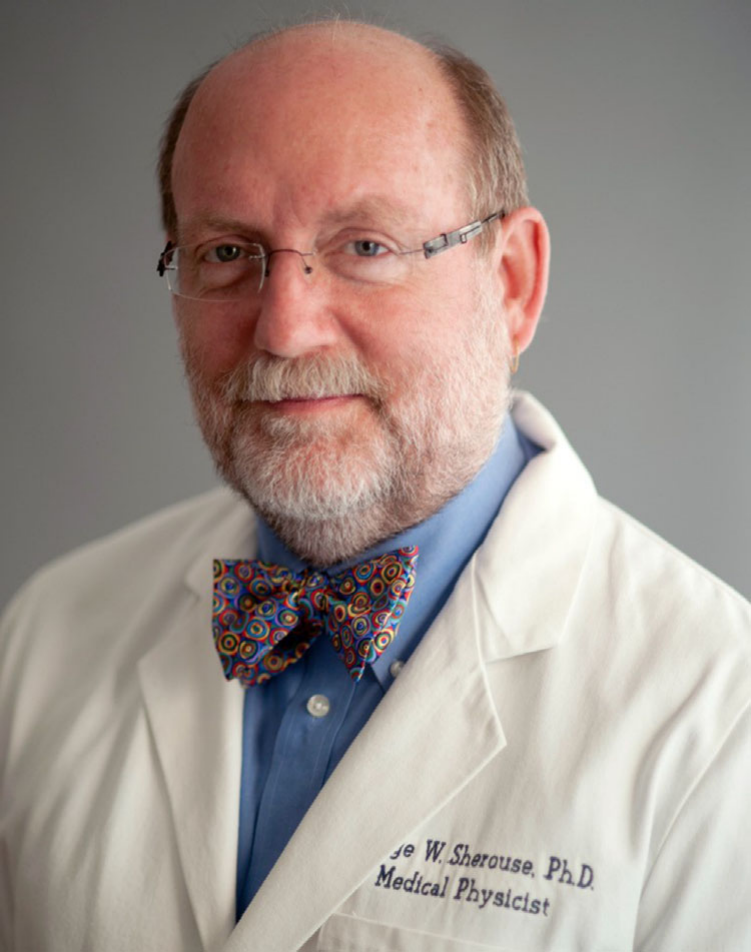


February 18, 1956–January 9, 2022

Dr. George Winthrop Sherouse, age 65, of Durham, North Carolina, passed away peacefully at home on January 9, 2022, following a sudden onset of cholangiocarcinoma. He made significant contributions to the field of medical physics and impressed upon his family, friends, and colleagues his irrepressible joy for life, insatiable curiosity, and boundless creative energy.

Dr. Sherouse would often refer to himself as a simple country physicist, but he was not simple. His contributions to the profession were significant and his skills as a clinical medical physicist were exceptional. Among his many contributions, Dr. Sherouse developed GRATIS, the first modern graphical user interface‐based three‐dimensional (3D) treatment planning system. He idealistically chose to freely share his software rather than commercialize it. He was instrumental in pioneering 3D virtual simulation techniques and technologies that are now considered to be the standard of care for radiotherapy treatment planning. Countless patients have benefited from Dr. Sherouse's contributions to modern radiotherapy planning.

In the profession and beyond, he was a man of strong convictions followed decisively. As he put it: "Those who embrace 'yes' make more choices than most, and some of those choices will be mistakes. If the choices are bold, then the mistakes can be big. But mistakes can also access rich opportunities for joy and growth. The Danish physicist and wag Niels Bohr is remembered as saying that an expert is someone who has made all the mistakes that can be made in a narrow field. Dr. Sherouse has expertise in many things."

Dr. Sherouse was born in Jacksonville, FL, on February 18, 1956, to Clarence and Ruth Tomlinson Sherouse. He grew up in Eau Gallie, FL in the bosom of the space program where his natural gifts of intelligence and curiosity were met with a remarkable public education. As a child, he was very interested in model rockets and photography. In high school, his physics teacher inspired him to continue in that direction.

He attended the University of Florida (UF) as an undergraduate, earning a B.S. in Physics in 1977. He was very interested in Computer Science, Philosophy, and other liberal arts. He entered the graduate program in Physics at UF where he failed to thrive. The graduate advisor was supportive of his finding a job and was able to recommend an opening in the physics division of the Radiation Oncology department at the hospital for a physics grad who could do some programming. Having become aware of the specialty, he ultimately entered the Medical Physics graduate program at UF and earned his M.S. in 1981 with a project to use a microcomputer to control a 3D beam scanning device. During his intensive clinical training at UF, he absorbed from his mentors a sustaining vision of the Medical Physicist as a technologically sophisticated clinical problem solver.

As the nominal computer guy in the department, he also had close interactions with the company that provided the treatment planning system, Atomic Energy of Canada Ltd. That connection led to a job offer and brought him to Ottawa, where Dr. Sherouse was fortunate to work closely with Jack Cunningham to sort out some knotty problems in the planning system code, provide tech support to a diverse customer base, and formulate his own vision for what treatment planning could be. In 1983, Dr. Sherouse became a Clinical Assistant Professor and Director of Computing Services in the Department of Radiation Oncology at North Carolina Memorial Hospital, University of North Carolina (UNC), Chapel Hill. There, he established the MEDPHYS email list for medical physicists, a version of which is still in regular use today. Dr. Sherouse was certified by the American Board of Radiology in Therapeutic Radiologic Physics in 1991. While at UNC, he spent nearly a decade developing GRATIS and the original virtual simulator. That work and its clinical implementation provided the dissertation for his Ph.D., awarded in Biomedical Engineering by UNC in 1992. In 1998, Dr. Sherouse left academic medical physics to focus on his own business as an independent contractor and consultant, primarily serving clients throughout Virginia and North Carolina.

Throughout his career, he authored peer‐reviewed journal articles, book chapters, and presented his work at national and international conferences. Dr. Sherouse was very active in the American Association of Physicists in Medicine (AAPM) and was especially engaged in the Professional Council on issues of ethics and professionalism. He served as the president of the Southeast Chapter of the AAPM in 1997. He was recognized as a Fellow of the AAPM in 2007. He co‐authored the 2009 and 2019 Code of Ethics, was extremely active in the Professional Economics Committee and served as a Board Member‐At‐Large for two different terms.

Through his presentations, Dr. Sherouse offered us his compelling and provocative stances on safety and quality, ethics, and professional identity. Dr. Sherouse could pinpoint the crux of any matter. His out‐of‐box thinking was extraordinary and honest. He was a mentor to many and inspired even more. His thought‐provoking conversations often inspired physicists to identify issues and Dr. Sherouse encouraged and empowered AAPM members to spearhead new initiatives. Many will miss his eloquent presentations of ideas.

One of Dr. Sherouse's proudest professional accomplishments was the creation of a medical physics residency program at Vassar Brothers Medical Center in New York. This program was one of the first community practice‐based residencies designed to provide formal clinical training for medical physicists. Throughout his career, he made it his mission to ensure that those under his instruction were well prepared to function as clinical Medical Physicists. He readily dispensed honest, reasoned, and sound advice to those who sought his counsel.

Those close to him benefited from his eclectic interests, penchant for adventure, and his pursuit of an authentic life over conformity. He had a distinctive personal style and a playfully unconventional approach to life. He was reserved but charismatic and enjoyed the lively company and new experiences. Music was a constant companion throughout his life, which he shared with others in many ways. His three sons carry forward his curiosity and creativity and exhibit also his deep love of music, travel, film, and photography.

He is survived by his beloved wife Caroline Sherouse, his brother Milton Sherouse, his first wife Liz Sherouse, and their two sons Perry Sherouse and Braxton Sherouse, his second wife Cathy Cole, his third wife Barbara Sparrow, and their son Hammond Sherouse.

This obituary was written in collaboration with Dr. Sherouse's family, friends, and colleagues. Dr. Sherouse's preliminary notes were a valuable starting point. Thank you to Dan Pavord, James Nunn, Per Halvorsen, Jeffrey Garrett, Sonja Dieterich, and James Goodwin for their insightful contributions.


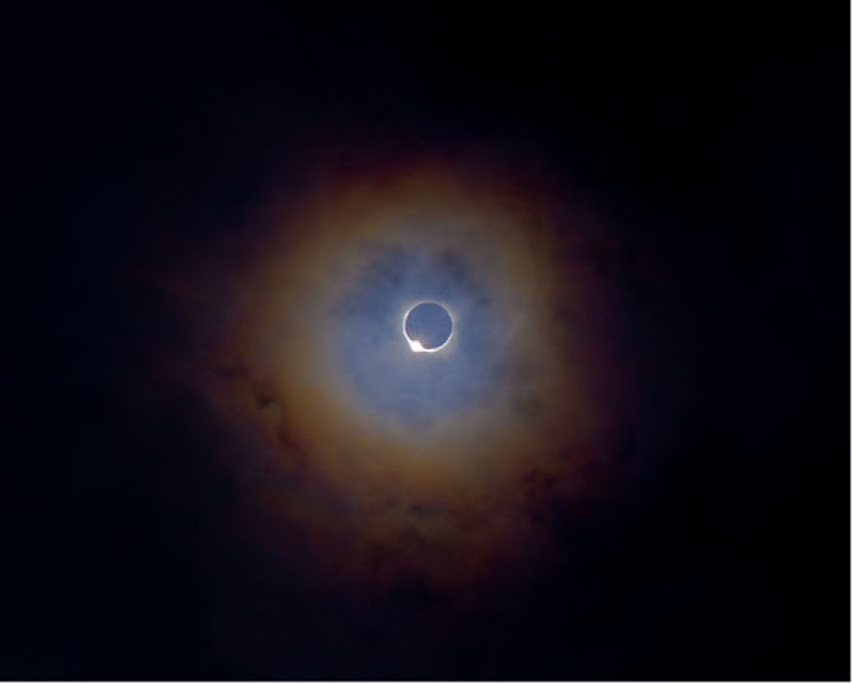


Dr. Sherouse became interested in photography as a kid and the hobby remained with him throughout his life. In 2017, he traveled to Folly Beach, South Carolina, with his son Hammond, to witness and photograph the ‘Great American Eclipse.’ Dr. Sherouse was looking forward to the next solar eclipse in 2024.

